# What are parents doing to reduce adolescent alcohol misuse? Evaluating concordance with parenting guidelines for adolescent alcohol use

**DOI:** 10.1186/s12889-015-1452-8

**Published:** 2015-02-10

**Authors:** Marie B H Yap, Anthony F Jorm, Dan I Lubman

**Affiliations:** School of Psychological Sciences, Monash University, Melbourne, Australia; School of Population and Global Health, University of Melbourne, Melbourne, Australia; Turning Point, Eastern Health and Eastern Health Clinical School, Monash University, Melbourne, Australia

**Keywords:** Prevention, Young people, Parenting, Internet, Alcohol consumption

## Abstract

**Background:**

The *Parenting Guidelines for Adolescent Alcohol Use* were developed to support parents in reducing adolescent alcohol misuse. The aims of this paper were to: (1) validate an online parent self-assessment survey as a criterion-referenced measure of parental factors that are important for predicting adolescent alcohol misuse; (2) examine parent web-users’ concordance with the *Parenting Guidelines* (extent to which their knowledge and behaviours align with *Guidelines* recommendations), and (3) examine the associations of parent and child characteristics with parental *Guidelines* concordance.

**Methods:**

Participants were 489 parents who completed the online survey. The survey assessed parent and child characteristics and parental concordance with the *Guidelines* in nine parenting areas. Reliability of the survey measure was assessed via an estimate of the agreement coefficient for each of the nine areas. Concurrent validity was examined by exploring the correlates of parental *Guidelines* concordance.

**Results:**

Reliability of the measure was acceptable to high in eight of the nine parenting areas. Greater parental *Guidelines* concordance was associated with being female, beliefs about healthy levels of drinking that align with the Australian national alcohol use guidelines, drinking within guidelines-recommended levels, the reduced likelihood of another adult in the household with a drinking problem, an older age of adolescent alcohol initiation, and greater confidence in the reported age of adolescent initiation.

**Conclusions:**

This validated self-assessment parenting measure can be useful for identifying targets for parenting interventions designed to prevent or reduce adolescent alcohol misuse, and as a pre- and post-intervention assessment to assess the effects of such interventions.

**Electronic supplementary material:**

The online version of this article (doi:10.1186/s12889-015-1452-8) contains supplementary material, which is available to authorized users.

## Background

Alcohol misuse amongst young people is a burgeoning public health concern. Prevention strategies that target the development of alcohol abuse and risky drinking during adolescence and early adulthood are particularly pertinent given the greater prevalence of these problems within this developmental period relative to other age groups [1].

When considering possible targets for preventative intervention, it is notable that adolescent drinking is influenced by a range of factors that parents can potentially control or modify [2], e.g. parental modelling and parent–child relationship quality. Consistent with this view, national guidelines for alcohol consumption have been published in both Australia and the United Kingdom. Both the *Australian Guidelines to Reduce Health Risks from Drinking Alcohol* released by the National Health and Medical Research Council [3] and *Guidance on the Consumption of Alcohol by Children and Young People* from the Chief Medical Officer of the United Kingdom [4] recommend that adolescents under the age of 18 should delay initiating alcohol consumption for as long as possible, and that those below 15 years not drink any alcohol at all. If adolescents aged 15–17 years consume alcohol, it should always be at a low risk level (within recommended adult limits) and in a safe environment, supervised by adults. In particular, both sets of guidelines are directed at parents and carers as implementers of the recommendations concerning underage drinking. However, they do not provide advice on how parents can do this. To support parents in implementing these guidelines with their child and hence reduce their risk for alcohol misuse and related harms, the Parenting Guidelines for Adolescent Alcohol Use [5] and a web-based parenting intervention [6] were developed and made readily accessible on the Parenting Strategies: preventing adolescent alcohol misuse website (www.parentingstrategies.net/alcohol) in 2011.

The *Parenting Guidelines* [5] were developed using a stringent and well-established methodology for developing various mental health related guidelines (e.g. [7-11]), which involved two stages. Firstly, to elucidate parenting factors to target for intervention, a systematic review of the evidence from longitudinal studies of parenting factors that predict delayed alcohol initiation and reduced levels of later drinking was conducted [2]. In order to translate this evidence into practical strategies that parents could employ to prevent or reduce their adolescent’s alcohol use, the second stage involved utilising the Delphi method to develop a set of expert consensus guidelines [10]. The resulting *Guidelines* provide 289 parenting strategies that were endorsed as important or essential in reducing adolescent alcohol use by ≥90% of the expert panel. Given that the *Guidelines* recommendations are supported by longitudinal evidence [2] and national experts [10], greater parental concordance with the *Guidelines* is expected to have a protective effect for the adolescent against alcohol misuse.

Despite the preponderance of longitudinal studies that have examined parental factors predicting adolescent alcohol misuse, the systematic review [2] revealed a paucity of well-validated standardised self-report measures of this whole range of parental factors. Consequently, we developed the Parenting Strategies online self-assessment parenting survey as a criterion-referenced measure, to assess parents’ knowledge and behaviours against our *Parenting Guidelines*. Once validated, this measure can be used to inform intervention strategies and targets by identifying areas of parental knowledge and practices that are not concordant with *Guidelines* recommendations. It can also be used to assess changes in parental *Guidelines* concordance when evaluating the effects of parenting interventions to prevent adolescent alcohol misuse.

The aims of this paper were three-fold: (1) to validate the online parent self-assessment survey as a criterion-referenced measure of parental factors that are important for predicting adolescent alcohol misuse; (2) to examine parental concordance with the *Parenting Guidelines*, as assessed by the survey, of parent web-users, and (3) to examine the associations of parental *Guidelines* concordance with parent and child characteristics. The validation of the survey includes examining its reliability and validity indicators. As a criterion-referenced measure, an indicator of its reliability is the agreement coefficient, which is a measure of the overall consistency by which examinees will be classified on two administrations of a mastery test [12]. The relevant validity indicators for this measure include face validity (at face value it seems to measure *Guidelines* concordance), construct validity (the variables assessing the various parenting areas converge onto the single proposed construct of overall concordance with *Parenting Guidelines*), concurrent validity (it can help to distinguish between parents who are doing better in reducing their adolescent’s risk for alcohol misuse from those who are not doing as well), and convergent validity (it should be correlated with other measures of parenting associated with adolescent alcohol misuse) [13]. The survey was designed to have face validity, and initial piloting with reference groups of parents confirmed this [6]. In the current study, the construct validity of the survey was examined by conducting a Principal Components Analysis (PCA) on the concordance variables for the various parental factors. Concurrent validity was examined by exploring the correlates of parental *Guidelines* concordance (which the survey is designed to measure). Likewise, convergent validity was also assessed by specifically examining its correlation with parental drinking, which is a major component of parental modelling of alcohol-related attitudes and behaviours addressed in the *Guidelines*.

## Methods

### Participants

Participants comprised self-selected web users who visited the www.parentingstrategies.net/alcohol website and completed the online self-assessment parenting survey. To be eligible for inclusion in this study, survey completers had to respond positively to questions asking whether they were “a parent who is interested in finding out information about adolescent alcohol use” and that their responses referred to an adolescent child.

Participants were recruited via a variety of means, including links to other parenting or mental health websites, promotional flyers sent through researcher networks, schools, parenting associations and other health organisations via a snow-balling technique (i.e. recipients were encouraged to pass the flyer on to any other interested parties via any means). Promotional materials appealed to parents concerned about protecting their adolescent child from alcohol-related problems, highlighting the availability of evidence and expert-endorsed parenting guidelines and a tailored parenting program on the www.parentingstrategies.net/alcohol website. Upon visiting the website, parents were invited to complete the self-assessment online survey in order to receive parenting strategies recommendations that are tailored to their individual situation (e.g. parents who report the absence of family rules receive tips about how to establish these with their adolescent). Prior to completing the survey, participants were informed that the information collected from them would be used for the purposes of research and evaluation, and their completion of the survey was taken to imply consent. Potential survey completers are presented with the definition of ‘adolescent’ as a young person aged 12–17 years, but users with children outside of that age range are also welcomed to complete the survey. This study was approved by the Human Research Ethics Committee of the University of Melbourne.

Between January 2011 and March 2014, the website received 9,840 unique visitors who engaged with the website content (e.g. read the Parenting Guidelines on the website or downloaded the Guidelines document). Of these visitors, 765 completed the survey, 503 of whom were parents (66%). Amongst the parent survey completers, 470 indicated that their responses to the survey referred to an adolescent who lives with them, while another 19 referred to another adolescent child (e.g. a non-resident parent reporting on their adolescent child). This resulted in a total sample of 489 parents included in the current study.

### Survey

The online survey was developed based on *Parenting Guidelines for Adolescent Alcohol Use* [5]. Details of the survey development have been published elsewhere [6] and will be briefly outlined here. The survey was designed as a parental self-assessment against the *Parenting Guidelines* and hence is a criterion-referenced measure. Specifically, the survey contains 111 items which assess parents for concordance to the recommendations of the *Guidelines* in nine parenting areas: (1) Knowledge about adolescent alcohol use; (2) Parent-adolescent relationship; (3) Talking about alcohol with their adolescent; (4) Parental modelling of alcohol-related attitudes and behaviours; (5) Establishing family rules and consequences; (6) Parental monitoring; (7) Preparedness for hosting adolescent parties (which may involve alcohol); (8) Involvement with their child’s peers and preparing their child for peer influence; and (9) Preparedness for handling adolescent alcohol misuse (see Table 1 for example items). An earlier draft of the survey was piloted with a reference group of 23 parents of adolescents to establish its face validity. Feedback from parents about the clarity of the items and response options was incorporated into the final version of the survey [6]. The purpose of the survey is to identify the areas of the user’s current parenting knowledge and practices which are falling short of (i.e. non-concordant with) the *Guidelines*’ standards. These aspects of the user’s parenting can then be targeted by the tailored online parenting program [6].

**Table 1:** **Guidelines topics covered in online survey, number of items, and example items and responses**

**Guidelines topic**	**Number of items**	**Example items**	**Response options**
Knowledge about alcohol	22		True/False
	-11 items about risks associated with adolescent drinking	- serious injury (true positive); hair turning grey at a younger age (false positive)	
	-11 items about factors that influence adolescents’ decision to drink	- to cope with stress (true positive); Adolescents think that alcohol goes well with fine food (false positive)	
Parent-adolescent relationship	10	I spend one-on-one time with my adolescent	Never/Rarely/Sometimes/Often
Talking about alcohol	2	Have you talked to your adolescent about the risks associated with alcohol?	No/No, my child is too young to need to know this/Not sure, maybe/Yes, I have and I think they have some awareness of the risks/Yes, I have and I think they are well aware of the risks
Modelling	10		
	-3 assessing beliefs (1 about parental supply and 2 about parental influence)	-Giving your adolescent an occasional alcoholic drink at home will help them learn to drink responsibly	-True/False
	-7 assessing behaviours	-[How often do you] get drunk at home?	- Never/Rarely/Sometimes/Often
Family rules	8		
	-2 assessing presence of rules (1 for general behaviour; 1 specific to alcohol)	- Have you set family rules specific to your adolescent’s use of alcohol?	Yes, definitely/Yes, partly/No, my child is too young to need rules about alcohol/No
	-6 assessing the process by which family rules are established and implemented	-Do you ever compromise on family rules?	-Not applicable, we don’t have family rules/No, family rules are non-negotiable/Yes, but only on minor matters/Yes, I bend the rules to appease my adolescent
Monitoring	5	When your adolescent is not with you, do you know where they are?	Never/Sometimes/Usually/Almost always
Adolescent parties	15	[If your adolescent was to host a party for their friends, how likely would you be to] leave them alone?	Very unlikely/Unlikely/Likely/Very likely
Involvement with child’s friends and managing peer influence	6	Have you talked with your adolescent about situations where they may feel pressure from peers to drink alcohol?	No/No, my child is too young to need to know this/Not sure, maybe/Yes, I have and I think they are somewhat prepared for such situations/Yes, I have and I think they are fully prepared for such situations
Adolescent alcohol misuse	7	[Have you talked to your adolescent about] the dangers of drink driving?	Yes/No

In addition, the survey collected information on demographics (age and gender of parent and child, country of residence, state of residence if in Australia, number of children in the household), parental drinking (three items adapted from the Alcohol Use Disorders Identification Test-Consumption (AUDIT-C) [14]), adolescent drinking (frequency, age of initiation, and parental confidence about these responses), parental concern about their child’s current alcohol use and risk for future alcohol and mental health problems, parental beliefs about healthy levels of drinking for an adult or an adolescent aged below 18, and the presence of other adults in the household with a drinking problem (See Additional file 1 for survey questions).

### Scoring of survey responses for guidelines concordance

In criterion-referenced tests, the cut-off score to indicate mastery of the skills of interest has to be set with caution, because test score interpretation and the validity thereof are directly dependent on the cut-off scores [15]. The cut-off scores to indicate mastery of or concordance with the *Guidelines*’ recommendations in the nine parenting areas were intentionally set to be very high for two reasons. Firstly, the *Guidelines’* recommendations for what parents should do were directly derived from longitudinal research evidence [2] and expert consensus [10], indicating their importance for preventing adolescent alcohol misuse. They are hence deemed as the gold standard to which parents should be encouraged to conform. Secondly, the survey serves as a pre-test for the online parenting program, to identify parents who could benefit from the intervention and hence obtain higher scores in the post-test. Specifically, the cut-off score was set at 100% accuracy or concordance in all but three of the nine parenting areas: (1) *Knowledge about alcohol* included some false positive items which were intentionally selected to be possibly true, but were not supported by existing research evidence. It was hence determined that some error (up to 20%) should be permitted; (2) *Parent–child relationship* items included suggestions for improving the parent–child relationship which were not necessarily prescriptive or independently predictive of adolescent drinking. It was hence determined that parents who complied with 9 out of 10 of the recommendations could be deemed concordant; (3) *Adolescent parties*, like *Parent–child relationship*, included some suggestions that may not apply in all situations. For instance, parents of young adolescents (e.g. 12-year-olds) may not have ever needed to check on the issue of alcohol before allowing their child to attend an adolescent party. Hence up to 20% non-compliance was determined to still reflect parental concordance. *Guidelines* concordance in each area was scored 1, with a total score that could range from 0 to 9.

### Data analysis

The data was first analysed using frequencies to examine parent and child characteristics. Descriptive statistics for scores on each of the nine parenting areas covered by the *Guidelines* were also examined.

The reliability of the criterion-referenced survey was assessed via an estimate of the agreement coefficient for each of the nine parenting areas following Subkoviak [12]. The agreement coefficient refers to the proportion of examinees consistently classified (as concordant or non-concordant) on two or more administrations of the same or parallel tests [12]. Subkoviak provided instructions and a table from which the agreement coefficient of a measure can be estimated using only a single test administration. He suggested that an agreement coefficient of 0.75 and above would constitute an acceptable value where half the examinees are masters and half non-masters, with higher values expected if the relative proportions of masters and non-masters become more dissimilar. Subkoviak also discussed the kappa coefficient as another measure of consistency, to indicate the gain in consistency realized by using the given test. Given that our aim was to assess the overall consistency by which parents would be classified as concordant with Guidelines or not, our analyses in this study focused only on the agreement coefficient.

To examine the construct validity of the survey measure, we conducted a Principal Components Analysis (PCA) with Varimax rotation, exploring both a one-factor solution and a multi-factor solution based on the scree plot.

To examine the concurrent validity of the survey measure and the correlates of parental concordance with the *Parenting Guidelines*, we first examined the bivariate correlations of the total concordance score and eight parent and six child characteristics variables. We then conducted a multiple linear regression to examine the associations that total concordance had with various parent and child characteristics variables (entered simultaneously). Parent characteristics included (1) age, (2) gender (coded as female = 0, male = 1), (3) concern about child’s current drinking, (4) concern about child’s risk for alcohol problems, (5) concern about child’s risk for mental health problems, (6) concordance of parental beliefs about healthy levels of drinking with Australian alcohol guidelines for adults and young people (coded as 1 = concordant on all three guidelines or 0 = not concordant on at least one guideline), (7) parental drinking (coded as 1 = within safe levels defined by Australian guidelines or 0 = above safe levels), and (8) the presence of other adults in the household with a drinking problem. The Australian alcohol guidelines recommend that a healthy adult should drink no more than two standard drinks (equivalent to 2.53 UK units) on any day and no more than four (5.07 UK units) on a single occasion of drinking [3]. These are broadly consistent with the UK guidelines, which recommend no more than 3–4 units a day for men and 2–3 for women [4].

Parent-reported child characteristics included: (1) age, (2) gender (female = 0, male = 1), (3) frequency of current drinking, (4) age of initiation, and parental confidence about responses regarding (5) drinking frequency and (6) initiation. Given that the age of initiation variable necessarily included a response ‘My child has never had alcohol to my knowledge’, which would result in missing values when the variable is computed as a continuous variable, we followed Cohen and Cohen’s [16] instructions for handling such missing values in order to include the full sample in analyses. Specifically, we created a dichotomous missing data variable and plugged the missing age-of-initiation values with the mean. Both of these variables were then included in the multiple regression analysis, but the statistics of the missing data variable were not reported.

All analyses were performed using PASW version 20. The *p* < 0.05 significance level was used.

## Results

### Sample characteristics

Parent and child characteristics are summarised in Tables 2 and 3 respectively. Most parents were aged between 40–59 years, female, and lived in Australia. Most parents expressed at least a little concern about their child’s risk of alcohol (83%) or mental health problems (75%), but less than half (48%) were concerned about their child’s current drinking. About 25% of parents reported the presence of other adults in the household with a possible or definite drinking problem. Seven in every ten parents reported beliefs about healthy levels of alcohol use that were in agreement with the Australian alcohol use guidelines. Sixteen years was the modal age of the target adolescent (the child who is the focus of the parent’s responses to the survey). Most target adolescents (84%) were aged between 12–17 years, and 48% were female. Based on parental report, less than 47% of the target adolescents had never drunk alcohol, and 31% had started drinking before age 15. Parental confidence ratings regarding these reports were quite high.

**Table 2:** **Descriptive statistics for parent characteristics (** ***N*** **= 489)**

**Parent Characteristics**	**Frequency**	**Percent**
*Age of parent*		
29 or younger	2	0.4
30–39	63	12.9
40–49	288	58.9
50–59	128	26.2
60–69	7	1.4
70+	1	0.2
*Gender of parent*		
Female	431	88.1
Male	58	11.9
*Country of residence*		
Australia	437	89.4
New Zealand	13	2.7
United States	11	2.2
Canada	4	0.8
United Kingdom	16	3.3
Other	8	0.4
*Australian State or Territory*		
Australian Capital Territory	11	2.2
New South Wales	78	16.0
Northern Territory	2	0.4
Queensland	41	8.4
South Australia	27	5.5
Tasmania	15	3.1
Victoria	234	47.9
Western Australia	41	8.4
I do not live in Australia	40	8.2
*Concern about child’s current drinking*		
Not at all	253	51.7
A little	111	22.7
Yes	75	15.3
Very much so	50	10.2
*Concern about child’s future risk of alcohol problems*		
Not at all	83	17.0
A little	212	43.4
Yes	132	27.0
Very much so	62	12.7
*Concern about child’s risk of mental health problems*		
Not at all	123	25.2
A little	181	37.0
Yes	110	22.5
Very much so	75	15.3
*Parent drinks within Australian guidelines recommendations*		
No	319	65.2
Yes	170	34.8
*Parental beliefs about healthy levels of drinking agree with Australian alcohol guidelines for adults and young people*		
No	146	29.9
Yes	343	70.1
*Presence of other adults in household with drinking problem*		
No	369	75.5
Not sure	55	11.2
Yes	65	13.3

**Table 3:** **Descriptive statistics for parent-reported child characteristics (** ***N*** **= 489)**

**Child Characteristics**	**Frequency**	**Percent**
*Age of child (in years)*		
11 or under	37	7.6
12	28	5.7
13	46	9.4
14	73	14.9
15	91	18.6
16	115	23.5
17	57	11.7
18 or over	42	8.6
*Gender of child*		
Female	236	48.3
Male	253	51.7
*Frequency of child’s drinking*		
Never	228	46.6
Monthly or less	117	23.9
2 to 4 times a month	95	19.4
2 to 3 times per week	30	6.1
4 or more times per week	19	3.9
*Parental confidence about reported frequency of child’s drinking*		
Not confident at all	17	3.5
A little confident	44	9.0
Somewhat confident	163	33.3
Very confident	265	54.2
*Child’s age of initiation*		
My child has never had alcohol to my knowledge	185	37.8
11 or under	26	5.3
12	15	3.1
13	48	9.8
14	62	12.7
15	88	18.0
16	48	9.8
17	15	3.1
18 or over	2	0.4
*Parental confidence about reported age of initiation*		
Not confident at all	9	1.8
A little confident	59	12.1
Somewhat confident	136	27.8
Very confident	285	58.3

### Reliability of criterion-referenced measure of parenting

Table 4 shows the descriptive and reliability statistics of parental concordance with the nine *Guidelines* topics. Reliability was mostly acceptable to high based on Subkoviak’s [12] estimates. The agreement coefficient was below the recommended 0.75 level in only one area, *Talking about alcohol* (0.70). Two-thirds of parents were concordant with *Guidelines* in the area of *Talking about alcohol*, but less than half showed concordance in the remaining eight parenting areas. Concordance was lowest for *Family rules* (7%) and *Modelling* (9%), followed by parental attitudes and behaviours regarding adolescent alcohol misuse. Total *Guidelines* concordance score was moderately low.

**Table 4:** **Descriptive and reliability statistics for mastery of Guidelines topics (** ***N*** **= 489)**

**Guidelines topic**	**Minimum**	**Maximum**	***M***	***SD***	**Highest possible total**	**Cut-off score**	**% mastery**	**z**	Cronbach’s alpha*	**Agreement coefficient**
Knowledge about alcohol^1^	0.00	1.00	0.65	0.24	1.00	0.80	39.10	1.42	0.69	0.91
Parent–child relationship	0.00	10.00	8.24	1.45	10.00	9.00	47.40	0.18	0.72	0.75
Talking about alcohol	0.00	2.00	1.53	0.71	2.00	2.00	65.60	0.04	0.62	0.70
Modelling	2.00	10.00	7.23	1.70	10.00	10.00	9.40	1.34	0.58	0.88
Family rules	0.00	5.00	2.94	1.09	8.00	5.00	7.20	1.44	0.43	0.88
Monitoring	0.00	5.00	3.67	1.16	5.00	5.00	22.90	0.71	0.57	0.77
Adolescent parties	3.00	15.00	11.87	2.87	15.00	12.00	46.20	0.13	0.80	0.80
Peers^2^	0.00	6.00	4.35	1.30	6.00	6.00	17.60	0.88	0.51	0.79
Adolescent alcohol misuse	1.00	7.00	4.82	1.57	7.00	7.00	11.90	1.07	0.64	0.88
*Total mastery score*	0.00	7.00	2.67	1.47	9.00					

*Note.* *Cronbach’s alpha is not regarded as a useful or meaningful measure of reliability in criterion-referenced testing [17]. They are provided above because they were computed as part of the process of estimating the agreement coefficient using Subkoviak’s [12] table. *Knowledge about alcohol* was scored as Proportion of true positives (out of 12) minus Proportion of false positives (out of 10), hence the highest total possible score was 1. *Peers* = Involvement with child’s friends and managing peer influence. z = the cut-off score of the Guidelines topic expressed as a standard score.

Further examination of the data on *Family Rules* revealed that while almost 95% of parents reported having rules for their adolescent’s behaviour and 76% were specific to alcohol, 87% did not involve their adolescent in developing such rules, 27% did not negotiate on family rules (when appropriate; i.e. on minor matters), 96% did not report adapting the rules to their adolescent’s maturity and responsibility, and 68% did not think that their adolescent had an adequate understanding of the rules.

Likewise, closer examination of the data on *Modelling* revealed that most parents (90%) correctly acknowledged their influence on adolescent drinking, but 32% believed that giving their adolescent an occasional alcoholic drink at home would teach responsible drinking. With regards to modelling of alcohol-related behaviours, although very few reported never declining an alcoholic drink (2%) or never using non-alcoholic ways of coping with stress (3%), many more parents reported behaviours that did not align with Guidelines recommendations. Specifically, 28% had never set and adhered to a limit for their own drinking, 45% had been drunk at home, 32% had driven after a few drinks, 51% had told amusing stories about someone getting drunk, and 73% had used alcohol at home to recover from a stressful day. Overall, half of the parents reported concordance with four or five (24% each) of the seven positive modelling behaviours, but only 12% reported concordance with all seven behaviours.

Finally, closer examination of the data on parental attitudes and behaviours regarding adolescent alcohol misuse revealed that 93% of parents recognised that even a single episode of binge drinking by adolescents is a matter of concern, and 86% had addressed the dangers of drink driving with their adolescent. However, substantially more parents had not addressed other related matters with their adolescent, including ways to remove themselves from situations where others are misusing alcohol (47%), the dangers of drink spiking (42%), and what to do if faced with a driver who had been drinking (33%). Moreover, 68% reported that if they found out that their adolescent had been misusing alcohol, they would either fail to address the matter calmly (11%), or would express their disappointment in their adolescent (rather than their specific behaviour; 64%), or both.

### Construct validity

We conducted a Principal Components Analysis (PCA) on the concordance variables for the nine parenting areas to examine the construct validity of the survey measure. The scree plot indicated that two factors should be retained for rotation, which accounted for 50.2% of variance. One factor comprised Talking about alcohol, Establishing family rules and consequences, Involvement with their child’s peers and preparing their child for peer influence (this loaded on both factors), and Preparedness for handling adolescent alcohol misuse. All four of these involve the parent talking with the adolescent about a particular issue (risks associated with alcohol, family rules, peer influence, possible situations where alcohol misuse may occur). The remaining five parenting variables loaded on the second factor. We also examined a single-factor solution, and this factor accounted for 30.4% of the variance, with factor loadings ranging from 0.369 to 0.722.

Given these findings, we opted for the single-factor solution for the following reasons: 1) there is no clear theoretical basis for separating the nine variables into the two factors indicated by the scree plot; it is likely that the four ‘talking’ variables converged because they all involved behaviours related to talking, rather than distinguishing between parenting factors associated with adolescent alcohol misuse per se; (2) the nine variables were created as part of a criterion-referenced measure, hence theoretically expected to comprise a single construct of overall concordance with the Parenting Guidelines; and (3) although the one-factor model accounts for less variance than the two-factor model, we deemed that 30.4% variance is acceptable.

Based on the above, we concluded that the construct validity of the factor reflecting overall parental concordance with the *Parenting Guidelines* is acceptable.

### Concurrent and convergent validity: correlates of guidelines concordance

Bivariate correlation analyses revealed that total parental concordance with the *Guidelines* was associated with all parent and child characteristics except for child age and gender (see Additional file 2). As presented in Table 5, findings from the multiple linear regression model were largely consistent with those from the correlation analyses. Greater concordance was associated with being a female parent, concordance of parental beliefs about healthy levels of drinking with the Australian alcohol use guidelines, parental drinking within guidelines-recommended levels, the absence (or reduced likelihood) of another adult in the household with a drinking problem, an older age of parent-reported adolescent alcohol initiation, and greater parental confidence in the reported age of adolescent initiation.

**Table 5:** **Findings from multiple linear regression examining the associations between total Guidelines mastery and parent and child characteristics (N = 489)**

	**Beta**	***t***	***p***
Parental age^1^	-0.05	-1.07	0.284
**Male parent**	**-0.09**	**-2.09**	**0.037**
Concern about adolescent’s current alcohol consumption	-0.06	-0.88	0.378
Concern about adolescent’s future risk of developing alcohol problems	-0.03	-0.52	0.604
Concern about adolescent’s risk of mental health problems	-0.01	-0.19	0.852
**Parental alcohol-related beliefs agree with Australian alcohol use guidelines**	**0.09**	**2.17**	**0.031**
**Parental drinking within safe levels according to Australian guidelines**	**0.16**	**3.88**	**0.000**
**Presence of other adults in household with drinking problem**	**-0.09**	**-2.20**	**0.028**
Child age^2^	0.10	1.65	0.099
Male child	-0.01	-0.22	0.830
Parent-reported frequency of adolescent drinking (current)	-0.13	-1.72	0.087
Confidence about reported frequency of adolescent drinking	0.07	1.27	0.203
**Parent-reported age of adolescent alcohol initiation**	**0.14**	**2.89**	**0.004**
**Confidence about reported age of adolescent alcohol initiation**	**0.16**	**3.00**	**0.003**
R^2^ = 0.22, *F* (15, 473) = 8.91, *p* < 0.001			

*Note.* ^1^Parent age was measured in years, within the categories 29 or under, 30–39, 40–49, 50–59, 60–69, and 70 or over. ^2^Child age was measured in years, within the categories 11 or under, 12, 13, 14, 15, 16, 17, and 18 or over. Significant predictors are presented in bold.

To examine post-hoc whether the children of parents who held guidelines-concordant beliefs about alcohol use were more likely to start drinking only after 15 years of age (as recommended in national guidelines), we conducted a descriptive analysis of the parent-reported age of alcohol initiation, amongst those whose parents held guidelines-concordant beliefs about alcohol use and reported that their adolescent had already started drinking. Forty-seven percent of these parents reported that their adolescent had started drinking before they turned 15 years.

### Sensitivity analyses

Given that the period of recruitment extended over three years, during which parental concordance with *Parenting Guidelines* may have changed, we computed a variable estimating the number of months in the interval between survey completion and data analysis to test this possibility. Bivariate correlation revealed that this variable was not correlated with total concordance (*r* = 0.08, *p* = 0.077). When the multiple linear regression model was run controlling for the interval variable, findings remained largely unchanged, except that the association between parent gender and parental concordance became non-significant, *β* =-0.09, *t* =-1.94, *p* = 0.053.

Likewise, given that the majority of our sample comprised Australian residents, we reran the multiple linear regression including only Australian residents to see if country of residence may be associated with concordance with Guidelines. As before, all significant findings remained unchanged except for parent gender, *β* =-0.07, *t* =-1.46, *p* = 0.144.

## Discussion

This study examined the reliability and construct, concurrent and convergent validity of a criterion-referenced measure of parental factors that predict alcohol initiation and levels of later drinking in adolescents. The reliability of the measure was acceptable to high for eight out of nine parenting areas, with *Talking about alcohol* just falling short of the recommended minimum on the agreement coefficient. This suggests that the overall consistency of the measure is acceptable. Findings also suggested that the construct, concurrent and convergent validity of the measure were acceptable.

### Parental concordance with parenting guidelines

Given that we set high cut-off scores to indicate mastery of the *Guidelines*, it is unsurprising that less than 50% of parents showed concordance with the *Guidelines* in all but one of the nine parenting areas—*Talking about alcohol*, for which two-thirds of parents were concordant. Nonetheless, concordance in two other areas—*Parent–child relationship* and *Adolescent parties*—approached 50%, and 39% showed concordance in *Knowledge about alcohol*. In contrast, it is notable that only 7% and 9% of parents showed concordance in the areas of *Family rules* and *Modelling* respectively. The low concordance rate for *Family rules* is particularly noteworthy given that this is also the area with the lowest relative cut-off score (62.5% accuracy). Further examination of the data revealed that parents most commonly showed non-concordance with *Guidelines* in the *process* by which family rules are established and implemented, e.g. level of involvement of the child in the development of the rules, adapting the rules to the child’s maturity and responsibility. Given the important influence that family rules and general discipline during adolescence have on adolescent alcohol misuse, especially on young people’s later drinking levels [2,18,19], these findings highlight the need to support parents in the process of establishing appropriate and reasonable boundaries for their adolescent’s behaviours, not just around the issue of alcohol but also more generally.

Low concordance in the area of *Modelling* is of concern given evidence from longitudinal studies that poor parental modelling of alcohol-related behaviours and attitudes predicts earlier alcohol initiation and higher levels of alcohol consumption by young people [2]. Closer examination of the data revealed that most parents (90%) correctly acknowledged their influence on adolescent drinking, but 32% did not agree with the guideline that parents can teach responsible drinking without providing alcohol to their adolescent at home. The latter may reflect a cultural belief that parents can teach responsible drinking through the provision of alcohol, which stands in stark contrast to research evidence that early initiation predicts more alcohol-related problems later in life [2,20]. Moreover, only 12% of parents reported concordance with all seven positive modelling behaviours. Specifically, the most problematic behaviours include: using alcohol at home to recover from a stressful day (portraying alcohol as a good way to deal with stress), telling amusing stories about someone getting drunk (conveying an idea that alcohol is fun or glamorous), and getting drunk at home, especially in front of their children. In a culture where alcohol is such an accepted and ingrained part of life, it is likely that parents are not conscious of the effects that some of these more subtle behaviours can have on their children. These findings highlight specific targets for future community-based education campaigns for parents.

Parental concordance with the *Guidelines* was also low in terms of attitudes towards adolescent alcohol misuse and preparing their adolescent for alcohol misuse by others (12%). Specifically, although most parents recognised the concerning nature of binge drinking and had addressed the issue of drink driving with their adolescent, one-third to almost half of parents had not addressed other related issues, including the adolescent removing themselves from situations involving alcohol misuse by others or their designated driver, and drink spiking. Furthermore, 68% of parents were not concordant with *Guidelines* in the way they would respond to alcohol misuse in their adolescent, especially in the way they expressed their disappointment (targeting the adolescent personally as opposed to their specific behaviour).

### Correlates of parental concordance with parenting guidelines

The significant correlates of parental concordance with the *Parenting Guidelines* that emerged from the simultaneous regression model support the concurrent validity of the online survey. Most importantly, it is promising to find that parents who were more concordant with the *Guidelines* reported that their adolescents started drinking at an older age, and were more confident in their report of age of initiation. There was also a non-significant trend with parent-reported frequency of current adolescent drinking, suggesting that more *Guidelines*-concordant parents were more likely to report less frequent or no drinking by their adolescent. The non-significance of the latter finding may be due to the difficulty for parents to monitor accurately the level of drinking by their adolescent, given that adolescents may intentionally conceal their drinking [21].

Parents who were more concordant with the *Parenting Guidelines* were more likely to hold beliefs about healthy levels of drinking that are concordant with all three of the Australian alcohol use guidelines, and to be drinking within safe levels according to these guidelines. The latter finding is noteworthy because it provides some support for the convergent validity of the survey, in suggesting that more *Guidelines*-concordant parents were indeed modelling more responsible drinking behaviours. Whilst it is promising that guidelines-concordant beliefs about healthy levels of alcohol use are associated with concordance with *Parenting Guidelines*, it is nonetheless noteworthy that beliefs did not necessarily translate into behaviours, with 70% parents holding beliefs that agree with the alcohol use guidelines, but only 35% drinking within the guidelines-recommended levels. Moreover, amongst parents who held guidelines-concordant beliefs about alcohol use and reported that their adolescent had started drinking, 47% reported that their adolescent had started drinking before the age of 15 years, which violates both the Australian and UK alcohol use guidelines that young people below 15 years should not drink at all.

In the current sample of parents, which comprised 88% mothers, being a female was associated with greater concordance with the *Parenting Guidelines*. However, given the small sample of fathers—a common finding in most studies involving parents [22], and findings from our sensitivity analyses that this finding was not robust—the implications of this finding are unclear. Finally, parents who were more concordant with the *Parenting Guidelines* were also less likely to report that there is another adult in the household with a drinking problem. This may reflect an overall family culture of responsible drinking.

### Limitations of the study

The findings from this study should be interpreted in light of its limitations. Firstly, the sample was self selected and predominantly female. Likewise, the parents were mainly from Victoria, Australia, where the Parenting Strategies website was developed. These limit the generalizability of the findings to fathers, to other regions of Australia and more broadly to other parents who had not self selected to use the website. It is also possible that some parent participants are researchers or other professionals (e.g. teachers, staff from education departments or health organisations) who completed the survey on their own children, given that our sampling strategies may have inadvertently targeted them. If this was the case, our reported findings may be an over-estimation of parental concordance with the *Guidelines*. The *Parenting Guidelines* were designed to complement the Australian alcohol use guidelines, and hence differences in recommendations across countries (e.g. legal drinking age) may limit the perceived relevance of the program in other countries. Nonetheless, the recommended parenting behaviours in the *Parenting Guidelines* are likely to apply similarly across many English-speaking countries, given that they were based on research evidence from international studies [2]. In particular, the program is likely to be relevant in the UK, given the similarity in recommendations for alcohol use by young people between Australia and UK. The cut-off scores for determining concordance with *Guidelines* were set based on expert judgement by the authors, but could be criticised as being rather arbitrary. However, the rationale for the cut-off scores was clearly provided and in light of the reliability and validity findings from the study, was deemed reasonable [15,23]. Finally, sole reliance on parent informants in this study precludes validation of the data with other sources, especially the adolescent. In particular, recent findings suggest that parents may be unaware of (and hence under-report) their child’s drinking, especially amongst younger adolescents [21].

## Conclusions

This study validated a self-assessment survey of parenting associated with adolescent alcohol misuse, demonstrating acceptable reliability and concurrent validity as a criterion-referenced measure, assessed against a set of *Parenting Guidelines* which are supported by longitudinal research evidence and expert consensus as important for preventing adolescent alcohol misuse. This measure can be useful for identifying targets for parenting interventions designed to prevent or reduce adolescent alcohol misuse, and as a pre- and post-intervention assessment to assess the effects of such interventions on important parental factors influencing adolescent risk for alcohol misuse.

## Additional files

Additional file 1:
**Survey questions.**
Additional file 2:
**Bivariate correlations.**
