# Supplementary material for: What are parents doing to reduce adolescent alcohol misuse? Evaluating concordance with parenting guidelines for adolescent alcohol use
Source: BMC Public Health. 2015 Feb 10;15:114. doi: 10.1186/s12889-015-1452-8 (PMC4331448; doi:10.1186/s12889-015-1452-8)
Supplement: Additional file 1: — Survey questions. [file 12889_2015_1452_MOESM1_ESM.docx]

Additional file 1: Survey Questions

# Your family

1. What is your main reason for completing this survey?

| I’m a parent who is interested in finding out information about adolescent alcohol use |
| --- |
| I’m a professional who is interested in finding out more about adolescent alcohol use |
| I’m neither a parent nor a professional but I’m interested in finding out more about adolescent alcohol use |
| I’m just checking out the website |

2. How many children under the age of 18 are there currently living in your household?

| 0 |
| --- |
| 1 |
| 2 |
| 3 |
| 4 |
| 5 or more |

3. How many children aged 18 or over are there currently living in your household?

| 0 |
| --- |
| 1 |
| 2 |
| 3 |
| 4 |
| 5 or more |

4. How many of these children are between the ages of 12 to 17?

| 0 |
| --- |
| 1 |
| 2 |
| 3 |
| 4 |
| 5 or more |

This survey asks you to provide information about your approach to parenting and one specific child, so that the feedback provided at the end of the survey will be more relevant to you and this particular child. If you have more than one child **please focus on one particular child when considering your responses**. After completing the survey for this child, you are welcome to complete the survey again for a different child if you wish to do so.

5. Please indicate the age of the adolescent child who is the focus of your answers to this survey:

| 11 or under |
| --- |
| 12 |
| 13 |
| 14 |
| 15 |
| 16 |
| 17 |
| 18 or over |

6. Please indicate the gender of your adolescent child:

⭘ Male ⭘ Female

7. Do you live with the adolescent who is the focus of your answers?

⭘ Yes ⭘ No

# Your adolescent’s use of alcohol

The following questions relate to your adolescent’s alcohol use. When we say “consume alcohol” we mean having anything **more than a sip** of an alcoholic drink.

8. How often do you think your adolescent consumes alcohol?

| Never |
| --- |
| Monthly or less |
| 2 to 4 times a month |
| 2 to 3 times per week |
| 4 or more times per week |

9. How confident are you about your response to the above question?

| Not confident at all |
| --- |
| A little confident |
| Somewhat confident |
| Very confident |

10. If your adolescent has consumed alcohol, how old do you think they were when they started?

| My child has never had alcohol to my knowledge |
| --- |
| 11 or under |
| 12 |
| 13 |
| 14 |
| 15 |
| 16 |
| 17 |
| 18 or over |

11. How confident are you about your response to the above question?

| Not confident at all |
| --- |
| A little confident |
| Somewhat confident |
| Very confident |

12. Are you concerned about your adolescent’s **current** alcohol consumption?

| Not at all |
| --- |
| A little |
| Yes |
| Very much so |

13. Are you concerned about your adolescent’s risk of developing alcohol problems **in the** **future**? (By “alcohol problems” we mean any drinking behaviour that leads to problems at work, school or home, damage to health, or to legal problems)

| Not at all |
| --- |
| A little |
| Yes |
| Very much so |

14. Are you concerned about your adolescent’s risk of mental health problems?

| Not at all |
| --- |
| A little |
| Yes |
| Very much so |

You and your use of alcohol

Please provide us with a small amount of information about yourself.

15. Your age:

| 20-29 |
| --- |
| 30-39 |
| 40-49 |
| 50-59 |
| 60-69 |
| 70+ |

16. Your gender:

⭘ Male ⭘ Female

17. Which country do you live in?

(drop down list; branch to (14) only if “Australia”, otherwise go directly to (15))

18. If Australia, which state or territory do you live in?

| ACT |
| --- |
| NSW |
| Northern Territory |
| South Australia |
| Tasmania |
| Victoria |
| Western Australia |

In order to understand your adolescent’s family situation we need to know a little bit about your own alcohol consumption.

19. How often do you have a drink containing alcohol?

| Never (skip to next section) |
| --- |
| Monthly or less |
| 2 to 4 times a month |
| 2 to 3 times per week |
| 4 or more times per week |

20. How many drinks containing alcohol do you have **on a typical day** **when you are drinking**?

(Put in table of drinks here)

| 1 or 2 |
| --- |
| 3 or 4 |
| 5 or 6 |
| 7, 8 or 9 |
| 10 or more |

21. How often do you have **4 or more drinks on one occasion**?

| Never |
| --- |
| Less than monthly |
| Monthly |
| Weekly |
| Daily or almost daily |

# Your opinions about alcohol use

Now that you have told us about your drinking and your adolescent’s drinking, we would like to ask you some questions about what you think are healthy levels of drinking.

22. How many alcoholic drinks **per day** do you think **a healthy adult** should limit themselves to, in order to minimise the risk of **alcohol-related health problems (ie injury, disease, or death) over a lifetime**?

| 0 |
| --- |
| 1 |
| 2 |
| 3 |
| 4 |
| 5 |
| 6 or more |

23. How many alcoholic drinks do you think **a healthy adult** should limit themselves to **per day,** in order to minimise the risk of **alcohol-related injury (ie, injuries that would require treatment in an emergency department at a hospital)**?

| 0 |
| --- |
| 1 |
| 2 |
| 3 |
| 4 |
| 5 |
| 6 or more |

24. What do you think is the **maximum number of alcoholic drinks per day a healthy adolescent (under the age of 18) can consume** if they want to minimise the risks associated with alcohol consumption?

| 0 |
| --- |
| 1 |
| 2 |
| 3 |
| 4 |
| 5 |
| 6 or more |

# Your Knowledge about Alcohol Use During Adolescence

Parents need to know about the risks associated with drinking during adolescence, and what factors influence adolescents’ decisions about alcohol. The next section assesses your knowledge in these areas.

25. Which of the following do you think are risks associated with drinking alcohol during adolescence?

|  | Y | N |
| --- | --- | --- |
| 25.1. Being the victim of violent attacks or sexual assault |  |  |
| 25.2. Becoming violent during confrontations |  |  |
| 25.3. Having problems with skin, e.g. acne (FP) |  |  |
| 25.4. Becoming an alcoholic in adulthood |  |  |
| 25.5. Hair turning grey at a younger age (FP) |  |  |
| 25.6. Developing a food allergy (FP) |  |  |
| 25.7. Developing a mental illness such as depression OR becoming suicidal |  |  |
| 25.8. Developing or aggravating asthma (FP) |  |  |
| 25.9. Dropping grades or dropping out of school |  |  |
| 25.10. Serious injury due to falls or road accidents |  |  |
| 25.11. Being more susceptible to colds and flu (FP) |  |  |

26. Which of the following factors do you think influence adolescents’ decision to drink alcohol?

|  | T | F |
| --- | --- | --- |
| 26.1. Adolescents think that drinking is an ‘adult’ thing to do |  |  |
| 26.2. Adolescents are attracted by the smell of alcoholic drinks (FP) |  |  |
| 26.3. Adolescents get a thrill out of taking risks and trying new things |  |  |
| 26.4. Adolescents think that alcohol goes well with fine food (FP) |  |  |
| 26.5. Adolescents drink to be accepted by their friends |  |  |
| 26.6. Adolescents like the taste of alcohol (FP) |  |  |
| 26.7. Movies and popular media make it look like drinking makes adolescents more attractive |  |  |
| 26.8. Adolescents drink to cope with stress |  |  |
| 26.9. Adolescents enjoy having a hangover (FP) |  |  |
| 26.10. Adolescents are more likely to drink if alcohol problems run in their family |  |  |
| 26.11. Adolescents find that alcohol gives them more energy (FP) |  |  |

# Alcohol consumption in the family

27. Please indicate whether you think the following statements are true or false:

|  | T | F |
| --- | --- | --- |
| 27.1. Giving your adolescent an occasional alcoholic drink at home will help them learn to drink responsibly (FP) |  |  |
| 27.2. Parents don’t need to worry if their adolescent binge drinks on one occasion, because binge drinking is common among adolescents. (FP) |  |  |
| 27.3. Parents’ attitudes towards alcohol, what they drink, how much, when and where they drink are all a major influence on how their adolescent will drink in the future. |  |  |
| 27.4. Even though peer influence increases during adolescence, parents can still have significant influence on their adolescent’s decisions about alcohol. |  |  |

28. Please indicate how often you do the following:

|  | Never; rarely; sometimes; or often | | | |
| --- | --- | --- | --- | --- |
| 28.1. Make a decision about how much I will drink and stick to it |  |  |  |  |
| 28.2. Choose not to drink when is offered |  |  |  |  |
| 28.3. Get drunk at home |  |  |  |  |
| 28.4. Drive after having had a few alcoholic drinks if I feel sober enough |  |  |  |  |
| 28.5. Tell amusing stories to my family about situations where someone has drunk too much alcohol |  |  |  |  |
| 28.6. Have an alcoholic drink at home to recover from a stressful day |  |  |  |  |
| 28.7. Use ways of coping with stress other than alcohol (such as exercise, listening to music, or talking things over) |  |  |  |  |

## 29. Are there any adults in your household (other than yourself) who you think may have a drinking problem? Yes/no/not sure

# Talking to your child about alcohol

30. Have you talked to your adolescent about the **risks associated with alcohol**?

| No |
| --- |
| No, my child is too young to need to know this |
| Not sure, maybe? |
| Yes, I have and I think they have some awareness of the risks |
| Yes, I have and I think they are well aware of the risks |

31. Have you talked to your adolescent **to prepare them for situations involving alcohol**?

| No |
| --- |
| No, my child is too young to need to know this |
| Not sure, maybe? |
| Yes, I have and I think they are somewhat prepared for such situations |
| Yes, I have and I think they are fully prepared for such situations |

32. Have you talked to your adolescent about…………….

|  | Y | N |
| --- | --- | --- |
| 32.1. different ways they may remove themselves from situations where others are misusing alcohol? |  |  |
| 32.2. the dangers of “drink spiking” and how they can protect themselves? |  |  |
| 32.3. the dangers of drink driving? |  |  |
| 32.4. what they should do if faced with a drink driver, such as phoning home to get picked up or using a taxi which you will pay for? |  |  |

# Family Rules

Now we are going to ask you some questions about family rules.

By **family rules** we mean any type of established expectations, limits or guidelines for your adolescent’s behaviour that your adolescent is aware of. F**amily rules** include rules for your adolescent’s behaviour in general, which may involve rules specific to alcohol use.

33. Have you set specific, defined rules regarding your adolescent’s **behaviour**?

| Yes, definitely |
| --- |
| Yes, partly |
| No |

34. Have you set family rules specific to your adolescent’s **use of alcohol**?

| Yes, definitely |
| --- |
| Yes, partly |
| No, my child is too young to need rules about alcohol |
| No |

35. Was your adolescent involved in developing family rules for them to follow?

| Yes, definitely |
| --- |
| Yes, partly |
| No |
| Not applicable – we don’t have family rules |

36. Do you ever negotiate with your adolescent on family rules?

| Yes, all the time |
| --- |
| Yes, but only on minor matters |
| No, family rules are non-negotiable |
| Not applicable – we don’t have family rules |

37. Do you ever review or change family rules?

| Yes, all the time |
| --- |
| Yes, to adapt to my adolescent’s maturity and responsibility |
| No, family rules apply for as long as my adolescent is under my care |
| Not applicable – we don’t have family rules |

38. How well do you think your adolescent understands these family rules?

| Very well |
| --- |
| Quite well |
| Not very well |
| Not at all |
| Not applicable – we don’t have family rules |

# When your adolescent is not with you

A normal and healthy part of adolescence is increasing independence. This could mean that your adolescent sometimes goes out without you.

39. When your adolescent goes out without you, do you:

|  | Never | Sometimes | Usually | Almost Always |
| --- | --- | --- | --- | --- |
| 39.1. know where they are? |  |  |  |  |
| 39.2. know what they are doing? |  |  |  |  |
| 39.3. know who they are with? |  |  |  |  |
| 39.4. let them tell you about their activities only if they want to? (FP) |  |  |  |  |
| 39.5. randomly check up on them to make sure that they are where they said they would be. (FP) |  |  |  |  |

# Your adolescent’s friends

Friends are a big part of your adolescent’s life. The following statements are about the contact you have with your adolescent’s friends:

|  | Yes | No |
| --- | --- | --- |
| 40.1. I talk to my adolescent about qualities that really count in a friend, such as being kind and trustworthy, rather than popular and “cool” |  |  |
| 40.2. When my adolescent’s friends come over I leave them alone (FP) |  |  |
| 40.3. I talk to my adolescent’s friends to get to know them better |  |  |
| 40.4. I talk to the parents of my adolescent’s friends to get to know their families better |  |  |
| 40.5. I let my adolescent know which of their friends I don’t like, and which of them I prefer my adolescent to spend time with (FP) |  |  |

## Your adolescent and pressure to drink alcohol from peers

Your adolescent may find themselves in situations where it is difficult for them to say no to alcohol, because of peer pressure.

41. How likely is it that your adolescent will be in situations where they will feel peer pressure to drink alcohol?

| Not likely |
| --- |
| Somewhat likely |
| Very likely |

42. Have you talked with your adolescent about situations where they may feel pressure from peers to drink alcohol?

| No |
| --- |
| No, my child is too young to need to know this |
| Not sure, maybe? |
| Yes, I have and I think they are somewhat prepared for such situations |
| Yes, I have and I think they are fully prepared for such situations |

43. How confident do you feel about talking with your child about situations where they may feel pressure from peers to drink alcohol?

| Not confident at all |
| --- |
| A little confident |
| Somewhat confident |
| Very confident |

## Adolescent Parties

Parties are important events in adolescents’ lives. The following questions ask about your adolescent and parties. If your adolescent has not been invited to or hosted a party before, please respond to the questions by considering what is likely in such situations.

44. If your adolescent **was invited to an adolescent party**, how often would you……..

|  | Never | Sometimes | Usually | Almost always |
| --- | --- | --- | --- | --- |
| 44.1. Obtain the name and number of the responsible adult supervising the party |  |  |  |  |
| 44.2. Contact the parent of the party host to find out if there will be alcohol allowed |  |  |  |  |
| 44.3. Contact the parent of the party host to confirm that the party will be adequately supervised |  |  |  |  |
| 44.4. Provide your adolescent with alcohol to take to the party (FP) |  |  |  |  |
| 45.4. Provide your adolescent with a small amount of alcohol to take to the party so that you know exactly how much they will be drinking (FP) |  |  |  |  |

45. If your adolescent **was to host a party for their friends**, how likely would you be to……………

|  | *Very unlikely to extremely likely* | | | |
| --- | --- | --- | --- | --- |
| 45.1. Be asked by your adolescent for permission to have the party? |  |  |  |  |
| 45.2. Look up at least one reputable “partysafe” website for advice? |  |  |  |  |
| 45.3. Leave them alone? (FP) |  |  |  |  |
| 45.4. Have a plan for dealing with gatecrashers? |  |  |  |  |

## Deciding whether to allow alcohol at parties for older adolescents (over 15 years of age)

46. The following statements are about **hosting parties for** **older adolescents**. Please indicate how strongly you agree or disagree with each statement:

|  | Strongly agree to strongly disagree | | | |
| --- | --- | --- | --- | --- |
| 46.1.Providing adolescents with alcohol at parties is okay providing that it is supervised (FP) |  |  |  |  |
| 46.2. It is okay to let adolescents drink alcohol at a party if they bring it themselves (FP) |  |  |  |  |
| 46.3. If allowing alcohol at a party for older adolescents, it is essential to let the parents of guests know |  |  |  |  |
| 46.4. If allowing alcohol at a party for older adolescents, it is important to make it clear to my adolescent and the guests that drunkenness will not be tolerated. |  |  |  |  |
| 46.5. I know the local/national laws regarding supplying alcohol to people under the legal drinking age |  |  |  |  |
| 46.6. I would not allow any alcohol at a party for older adolescents that I am hosting. |  |  |  |  |

# When your adolescent has been misusing alcohol

47. If I find out that my adolescent has been misusing alcohol…….

|  | Strongly agree to strongly disagree | | | |
| --- | --- | --- | --- | --- |
| 47.1. I would use it as an opportunity to calmly discuss my expectations for them around alcohol |  |  |  |  |
| 47.2. I would let my child know in no uncertain terms how disappointed I am in them (FP) |  |  |  |  |

***Your relationship with your adolescent child***

48. The following questions relate to your day-to-day interactions with your adolescent:

|  | Never | Rarely | Sometimes | Often |
| --- | --- | --- | --- | --- |
| 48.1. I spend one-on-one time with my adolescent |  |  |  |  |
| 48.2. I tell my adolescent that I love them |  |  |  |  |
| 48.3. I help my adolescent to cope with their disappointments |  |  |  |  |
| 48.4. I encourage my adolescent to discuss their problems and concerns with me |  |  |  |  |
| 48.5. I discourage my adolescent from taking on goals that have the potential to be extremely stressful (FP) |  |  |  |  |
| 48.6. I praise my adolescent for their efforts as well as their achievements |  |  |  |  |
| 48.7. I tease my adolescent about their mistakes to build their resilience (FP) |  |  |  |  |
| 48.8. I find ways for my adolescent to be involved in family life, such as doing chores or caring for brothers and sisters |  |  |  |  |
| 48.9. I let my adolescent decide how much they want to be involved in family activities, because it is important that they become independent (FP) |  |  |  |  |
| 48.10. We eat dinner together as a family |  |  |  |  |
| 48.11. I encourage my adolescent to express their opinion in everyday conversations |  |  |  |  |

You have now reached the end of the survey.

49. My answers to this survey have referred to:

| An adolescent child who lives with me |
| --- |
| Another adolescent child |
| Not a real child, I’m just testing out how the survey works |

Please press the button below to receive your feedback.
