# Supplementary material for: What are parents doing to reduce adolescent alcohol misuse? Evaluating concordance with parenting guidelines for adolescent alcohol use
Source: BMC Public Health. 2015 Feb 10;15:114. doi: 10.1186/s12889-015-1452-8 (PMC4331448; doi:10.1186/s12889-015-1452-8)
Supplement: Additional file 2: — Bivariate correlations. [file 12889_2015_1452_MOESM2_ESM.docx]

# Additional file 2

## Bivariate correlations amongst variables of interest (*N* = 489).

|  | | 2 | 3 | 4 | 5 | 6 | 7 | 8 | 9 | 10 | 11 | 12 | 13 | 14 | 15 |
| --- | --- | --- | --- | --- | --- | --- | --- | --- | --- | --- | --- | --- | --- | --- | --- |
| 1 | Total Guidelines concordance | -.04 | -.18^**^ | -.22^**^ | -.15^**^ | -.12^**^ | .20^**^ | .21^**^ | -.13^**^ | .01 | -.03 | -.22^**^ | .24^**^ | .25^**^ | .30^**^ |
| 2 | Parental age^1^ | 1 | .16^**^ | .03 | .06 | .05 | .01 | .02 | .02 | .21^**^ | .00 | .11^*^ | -.07 | .16^**^ | -.02 |
| 3 | Male parent |  | 1 | .00 | -.02 | -.04 | -.23^**^ | -.06 | -.01 | -.08 | .13^**^ | .02 | -.07 | -.08 | -.14^**^ |
| 4 | Concern about adolescent’s current alcohol consumption |  |  | 1 | .51^**^ | .42^**^ | -.08 | .03 | .06 | .44^**^ | .04 | .74^**^ | -.45^**^ | .06 | -.38^**^ |
| 5 | Concern about adolescent’s future risk of developing alcohol problems |  |  |  | 1 | .62^**^ | -.02 | .04 | .10^*^ | .07 | .13^**^ | .34^**^ | -.19^**^ | -.08 | -.13^**^ |
| 6 | Concern about adolescent’s risk of mental health problems |  |  |  |  | 1 | .01 | .03 | .02 | .07 | .05 | .33^**^ | -.16^**^ | -.06 | -.11^*^ |
| 7 | Knowledge of Australian alcohol use guidelines |  |  |  |  |  | 1 | .10^*^ | -.09^*^ | -.02 | -.04 | -.16^**^ | .11^*^ | .08 | .13^**^ |
| 8 | Parental drinking within safe levels according to Australian guidelines |  |  |  |  |  |  | 1 | -.03 | .07 | .04 | -.07 | .03 | .09 | .07 |
| 9 | Presence of other adults in household with drinking problem |  |  |  |  |  |  |  | 1 | .00 | -.01 | .12^**^ | .04 | -.01 | -.02 |
| 10 | Child age^2^ |  |  |  |  |  |  |  |  | 1 | .00 | .59^**^ | -.27^**^ | .65^**^ | -.23^**^ |
| 11 | Male child |  |  |  |  |  |  |  |  |  | 1 | .00 | -.05 | .12^*^ | -.07 |
| 12 | Frequency of adolescent drinking (current) |  |  |  |  |  |  |  |  |  |  | 1 | -.33^**^ | .17^**^ | -.30^**^ |
| 13 | Confidence about reported frequency of adolescent drinking |  |  |  |  |  |  |  |  |  |  |  | 1 | -.03 | .64^**^ |
| 14 | Age of adolescent alcohol initiation |  |  |  |  |  |  |  |  |  |  |  |  | 1 | .07 |
| 15 | Confidence about reported age of adolescent alcohol initiation |  |  |  |  |  |  |  |  |  |  |  |  |  | 1 |

*Note.* ^1^Parent age was measured in years, within the categories 29 or under, 30-39, 40-49, 50-59, 60-69, and 70 or over. ^2^Child age was measured in years, within the categories 11 or under, 12, 13, 14, 15, 16, 17, and 18 or over. **p* < 0.05; ***p* < 0.01.
